# Stimulation of the dorsolateral prefrontal cortex impacts conflict resolution in Level-1 visual perspective taking

**DOI:** 10.3758/s13415-020-00786-5

**Published:** 2020-05-06

**Authors:** Adam W. Qureshi, Laura Bretherton, Bethany Marsh, Rebecca L. Monk

**Affiliations:** Department of Psychology, Edge Hill University, Ormskirk, L39 4PY England

**Keywords:** Visual perspective-taking, Theory of mind, Executive function, Transcranial magnetic stimulation, Dorsolateral prefrontal cortex

## Abstract

Theory of mind is the ability to understand others’ beliefs, mental states, and knowledge. Perspective-taking is a key part of this capacity, and while previous research has suggested that calculating another’s perspective is relatively straightforward, executive function is required to resolve the conflict between the self and that other perspective. Previous studies have shown that theory of mind is selectively impaired by transcranial magnetic stimulation (TMS) of the dorsolateral prefrontal cortex (DLPFC). However, it has been hitherto unclear as to which specific aspect of perspective-taking is impacted. The current study administered rTMS (N = 31 adult participants) to the DLPFC (active condition) and vertex (control condition) in a within-subjects design. Participants completed a L1 VPT task after each stimulation session, and focus (relative performance on self-perspective trials compared with other perspective trials) and conflict indices (relative ability to resolve competing self/other perspectives) were calculated. Results showed that stimulation of the DLPFC selectively impaired the conflict index, suggesting that the DLPFC may be causally related with the resolution of conflict between self and other perspectives, and that self-other interference may rely on domain-general processes.

## Introduction

The ability to understand that others have different mental states, beliefs, knowledge, and intentions is referred to as Theory of Mind (Premack & Woodruff, 1978). Within this, Level-1 visual perspective-taking (L1 VPT) capacity is the understanding that, in the same situation, what you can see may differ from what another can see (McHugh, Barnes-Holmes & Barnes-Holmes, 2004; Moll & Tomasello, 2006). In order to measure these capacities, tasks generate a distinction between the perspective of the participant and an “other,” with respondents tasked with choosing their response based on the perspective cued (self or other; perspective selection), as well as calculating what can be seen by the “other” (Samson et al., 2010). A key L1 VPT task (Samson et al., 2010), the “dot task,” comprises conditions that assess the effect of perspective (taking the self or other perspective) and consistency (whether these perspectives are consistent or inconsistent). More recent work using this task also has developed measures of people’s ability to deal with “conflict,” where performance is compared between trials where the perspectives are inconsistent and trials where the perspectives are consistent (termed the conflict index; Bukowski & Samson, 2017) and focus (where performance is compared between trials where the self-perspective is taken and trials where the other perspective is taken).

Explorations in this area suggest that perspective calculation is relatively automatic and that the ability to handle competing perspectives (and select the correct one) is reliant on executive control (Qureshi, Apperly & Samson, 2010; Qureshi & Monk, 2018; Qureshi, Monk, Samson & Apperly, 2019). There also has been extensive research into the neural basis of perspective taking and for dealing with self/other inference and has been extensive. For example, earlier work by Stuss, Gallup, and Alexandra (2001) found that lesions to the right frontal lobe^1^ were associated with impaired visual perspective-taking abilities, suggesting that this brain area plays an important role in the ability to successfully infer different perspectives. Imaging studies using fMRI also have found that the right inferior frontal gyrus (IFG) and dorsolateral prefrontal cortex (dlPFC) are differentially activated when viewing the orientation of an actor in an egocentric and altercentric condition respectively (Mazzarela, Ramsey, Conson & Hamilton, 2013) and another found domain-specific activation in the right temporo-parietal junction (rTPJ), ventral medial prefrontal cortex, and ventral precuneus during a L1 VPT task (Schurz et al., 2015). A recent review by Bukowski (2018) also highlights the involvement of dlPFC, the posterior middle frontal gyrus, the IFG, the dorsal posterior parietal cortex, the TPJ, the intraparietal sulcus, the inferior posterior temporal cortex, and superior cerebellum in varying aspects of VPT (although literature was found to be less supportive of the role of the posterior precuneus in VPT ). Furthering this understanding, EEG studies demonstrate that the temporo-parietal areas and the right prefrontal cortex show increased activation during a VPT task (Beck, Rossion & Samson, 2018). As such, a growing body of evidence suggests that there is an extensive neural network associated with various aspects of VPT, namely the rIFG, dlPFC, and rTPJ, as well as right prefrontal cortex.

Despite the relative proliferation of imaging studies on perspective-taking and ToM, research has offered an inconsistent picture as to the key neural underpinnings of these capacities (see Bukowski, 2018). Furthermore, such research is relational and is therefore vulnerable to questions as to the causal connection between neural activation and behavioural outcomes (Weber & Thompson-Schill, 2010). It is here that transcranial magnetic stimulation (TMS) can be deployed fruitfully to advance discussions surrounding ToM and perspective taking. For example, administration of TMS to the rTPJ was found to be related to increases in self-other interference (Wang, Callaghan, Gooding-Williams, McAllister & Kessler, 2016). Similarly, in contrast to those in the sham stimulation condition, TMS to the rTPJ substantially increased response times when dealing with false beliefs, and substantially worsened accuracy in taking the other perspective (Costa, Torriero, Oliveri & Caltagirone, 2008). This suggests that rTPJ may play an important role in the resolution of conflict between self/other representations and handling false beliefs. Furthermore, a study by Kalbe et al. (2010) found that repetitive TMS (rTMS) of the right dorsolateral prefrontal cortex (rDLPFC) selectively impaired cognitive (but not affective) ToM (Kalbe et al., 2010).^2^ Cumulatively, this TMS research suggests that specific aspects of ToM and VPT are related to the rTPJ, although this may be due to a more general process of conflict or interference resolution rather than a more specific process (Darda & Ramsey, 2019). Nevertheless, it remains unclear as to which facets of cognitive ToM are related to the rDLPFC.

TMS research also may offer insights into ongoing debates regarding automatic imitation and inhibition. Observing another’s action results in a tendency to *automatically* imitate that action, and this propensity must be controlled if it is not required by the situation or current task (Brass, Bekkering, Wohlschläger & Prinz, 2000). Research has shown that training on automatic imitation improved performance on the Director task (that requires ToM), whereas training on generic inhibition did not (Santiesteban, White, Cook, Gilbert, Heyes & Bird, 2012). This suggests that inhibition of automatic imitation and ToM both involve a level of control over self-other representations and that these processes are not the same as those in “conventional” inhibition tasks (ibid).

This distinction has been supported by studies showing that automatic imitation and ToM rely on regions of the medial prefrontal cortex and temporo-parietal cortex that are distinct from more lateral prefrontal brain regions that are implicated in nonsocial executive control (Bardi, Six & Brass, 2017; Brass, Ruby & Spengler, 2009; Brass, Derrfuss & von Cramon, 2005; Wagner, Maril, Bjork & Schacter, 2001). A lesion study (Samson, Houthuys & Humphreys, 2015) also found that self-perspective inhibition deficits were not explained by general executive control issues. As such, there is evidence to support the assertion that self/other interference involved in the inhibition of automatic imitation and ToM is domain-specific, and distinct from domain-general executive functions (e.g., nonsocial executive control).

However, other studies have found that automatic imitation uses brain regions that are not associated with any specific ToM network (e.g., medial prefrontal cortex and rTPJ; Darda & Ramsey, 2019; Darda, Butler & Ramsey, 2018) and also that automatic imitation may not be related to social processes such as empathy (Cracco et al., 2018) and mimicry Genschow et al., 2017). This is in addition to evidence that domain-general executive functions are involved in ToM, and specifically in self-other control (Qureshi, Monk, Samson & Apperly, 2019). There also is converging evidence from brain imaging and stimulation studies which indicates that the inferior frontal gyrus (IFG), an area often associated with generic inhibitory control, is involved in dealing with self-other conflict in visual perspective-taking. In other words, inhibitory control appears to be involved in managing self-other conflict when it arises during visual perspective-taking (McCleery et al., 2011; Ramsey et al., 2013). It therefore may be suggested that the capacity to manage self-other interference, involved in both perspective-taking and ToM, is domain-general (although see Ramsey, 2018 for a discussion of reaction time measures in this area) as opposed to domain-specific.

In short, while there is research which suggests that automatic imitation and ToM rely in part on domain-specific processes, findings also suggest that ToM relies on more domain general processes (Qureshi et al., 2019; Qureshi, Samson, Apperly, Braithwaite, Andrews, and Bodley Scott, 2010). Because the DLPFC has been shown to be associated with such domain general processes, such as nonsocial processing and selection (Wagner, Maril, Bjork & Schacter, 2001), its involvement in self-other interference control also may be postulated. However, such assertions are currently speculative, because there has been a paucity of research examining the effect of disrupting the DLPFC on performance during tasks requiring the management of self-other interference.

The current research therefore constitutes a first step towards examining the role of the rDLPFC in L1 perspective-taking and, more pertinently, whether the domain-general role of the DLPFC in selection applies to L1 perspective-taking. Specifically, using continuous theta burst stimulation (cTBS), it will examine self/other interference (conflict index) and relative performance in taking another’s perspective (focus index) in a L1 VPT task.^3^ This will allow the causal role of the rDLPFC in VPT to be ascertained. Based on prior literature relating executive control to these indices (Qureshi et al., 2019), we predicted the following in light of the fact that rDLPFC stimulation significantly inhibits (reduces) the size of the motor evoked potential (MEP) in this area (Huang et al., 2005).

The conflict index will be affected by rDLPFC stimulation, while the focus index will not be affected by rDLPFC stimulation. Based on prior literature, which suggests that the DLPFC has a domain-general role in selection, these findings would show that role also applies to VPT, in particular the conflict index.

However, if rDLPFC stimulation affects both indices, a general role for the rDLPFC in VPT would be more likely, whereas if no effect of stimulation is shown on either index, an argument may be made for VPT being domain-specific.^4^

## Methods

### Participants

A total of 31 participants^5^ completed the study (15 females; mean age 22.53 (SD = 3.07); 27 right-handed), with no exclusions. No renumeration was received for taking part. Consent was obtained according to the Declaration of Helsinki, and ethical approval was gained from the Departmental Research Ethics Committee.

### Stimuli

**Fig. 1:**
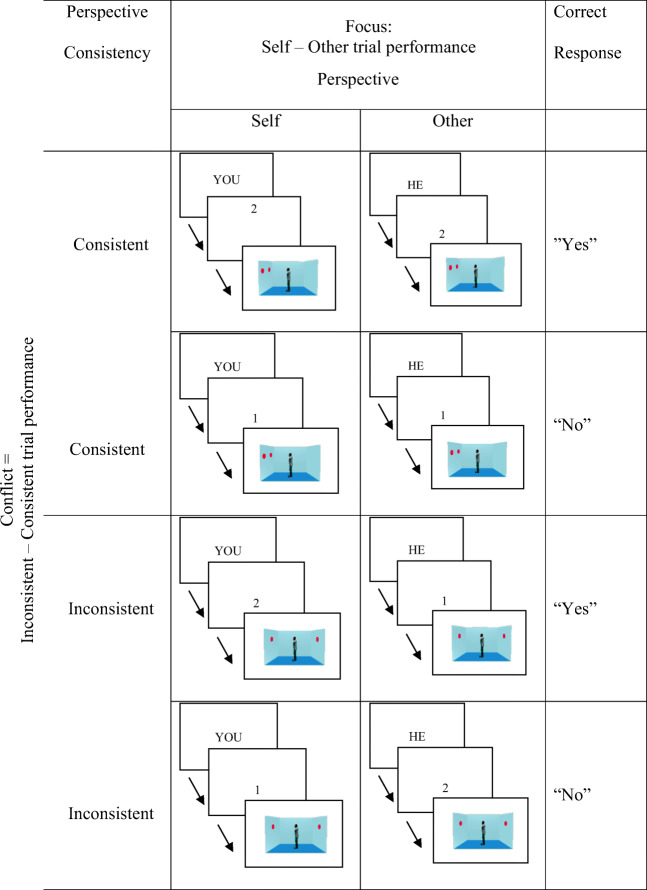
L1 VPT trial conditions

The L1 VPT task used the stimuli from Experiment 1 in Samson et al. (2010). These consisted of a picture displaying a lateral view into a room with the right, back, and left walls visible and with red discs presented on one or two walls (stimuli were created using the 3D animation program Poser 6, © Curious Lab). A centrally positioned human avatar faced either the right or left wall (Fig. 1). For half of the trials, the avatar’s position meant that he or she saw the same number of discs as the participants (Consistent condition), whereas for the other half, he or she could not see some of the discs that were visible to the participants (Inconsistent condition). For half of the trials, participants were asked to judge the number of discs from their own perspective (self-condition), and half from the avatar condition (other condition). Before the picture of the room, participants were cued as to which perspective to take (their own (you) or the avatar (he/she), and then as to how many discs they or the avatar could see (0/1/2/3). They were then presented with the picture of the room and were required to decide whether the information from the cues matched what the picture showed. For example, in first row of Fig. 1 (L), participants are asked to judge whether they could see two discs—based on the picture then presented, the correct response was “yes.” The gender of the avatar was matched to that of the participant. There was a total of 208 experimental trials presented in four blocks of 52 trials each. The order of trials and blocks was randomised (Qureshi & Monk, 2018, Qureshi, Apperly & Samson, 2010), and the task was presented using E-Prime 2.0.

### Design

A within-subjects design was used, with all participants completing the L1 VPT task after each stimulation session. The stimulation condition was the independent variable (control (vertex) x active (DLPFC)), with the Focus and Conflict indices as the dependent variables. Positive values in the Focus index indicated better performance in taking the other person’s perspective than the self-perspective (more altercentric rather than egocentric) and for the Conflict index, positive values indicated greater difficulty in handling conflicting perspectives.

### Procedure

Before taking part, participants completed the medical screening questionnaire to check for any excluding criteria (Rossi, Hallett, Rossini & Pascual-Leone, 2011). If they were able to take part, two simulation testing sessions were arranged, with a minimum of 3 days between them. The order of stimulation (control and active) was counter-balanced between participants.

### TMS

cTBS was performed using a 70-mm figure-of-eight stimulation coil (Magstim D70^2^ Coil), connected to a Magstim SuperRapid 2 Stimulator (The Magstim Company, Carmarthenshire, Wales). This produces a magnetic field of up to 0.8 T at the coil surface. To appropriately select the TMS stimulation intensity for each participant, the resting motor threshold (rMT^6^) for the first dorsal interosseous muscle (FDI) of the participant’s dominant hand was visually determined (Pridmore, Fernandes Filho, Nahas, Liberatos, and George, 1998). Here, the coil was positioned over the left or right motor cortex (for right or left-hand dominance respectively) in correspondence with the optimal scalp position (OSP). It was detected by moving the intersection of the coil in 1-cm steps around the motor hand area of the left motor cortex, while delivering TMS pulses at constant intensity. The coil was positioned tangentially to the scalp, at 90° from the midsagittal line, to modulate contralateral M1 excitability (Del Olmo et al., 2007) and thus assist with the detection of the rMT. The rMT was defined as the lowest stimulus intensity able to evoke a visible finger twitch on at least five of ten trials.

Active cTBS was delivered over the rDLPFC. The vertex was chosen as a control site to account for nonspecific effects of TMS. The approximate locations of the stimulation areas were identified on each participant's scalp by means of the international 10-20 EEG System Positioning. In keeping with past research (e.g., McNeill et al., 2018; Isegar, Padberg, Kenemans, Gevirtz & Arns, 2017), the coil was positioned on the F4 location for rDLPFC stimulation. With respect to the Montreal Neurological Institute (MNI) brain, F4 has been estimated to be approximately located at the following coordinates: 40.2 (x), 47.6(y), and 32.1 (z) (Okamoto et al., 2004). This lies within the right middle frontal gyrus, Brodmann area 46, and the Fronto-Parietal Control Network (Yeo et al., 2011).

Three-pulse bursts at 50 Hz repeated every 200 ms for 40 s were delivered at 80% of the subject’s resting MT, resulting in 600 pulses in total (Huang, Edwards, Rounis, Bhatia, & Rothwell, 2005). The inhibitory effect of cTBS on the MEP of the rDLPFC with this protocol lasts up to 30 minutes (Huang et al., 2005).

### L1 VPT task

After each stimulation session, participants completed the L1 VPT task. In accordance with Samson et al. (2010), participants were briefed on the task, and the gender of the avatar matched to that of the participant. Participants first completed 10 practice trials, followed by 4 blocks of 52 experimental trials with breaks between each block. The order of blocks was randomised, as were the trials within each block. The task took approximately 25 minutes to complete. Upon completion of both sessions, participants were thanked for their time and fully debriefed.

### Analytical procedure

Response time outliers (±2.5 SDs) were removed by condition and for each participant individually. Inverse efficiency scores were calculated for each condition (Response time/1 – error rate^7^), and indices of Conflict (inconsistent – consistent perspectives) and Focus (self-perspective – other perspective) were calculated as per Bukowski and Samson (2017). Higher inverse efficiency scores equate to higher processing costs, as a larger error rate will inflate the response time value.

## Results

The mean rMT was 67.90 (*SD* = 11.63), and this did not differ between right and left-handed participants. The mean number of days between experimental sessions was 4 (minimum of 72 hours).

### Focus and Conflict Indices

**Fig. 2:**
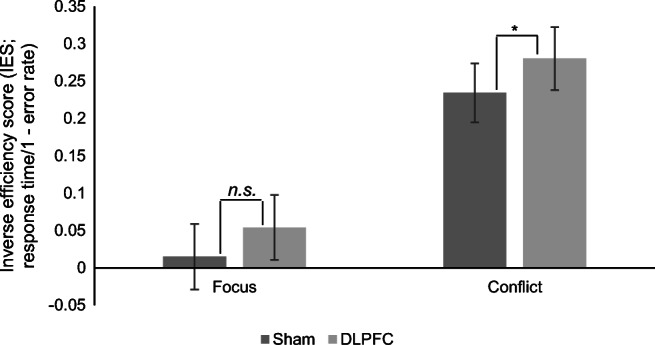
Focus x Conflict Indices by stimulation location (bars = confidence intervals; *p < 0.05, **p < 0*.01)*

Two separate repeated-measures ANOVAs were used to analyse the effect of stimulation of the rDLPFC (relative to that of the vertex) on Focus and Conflict indices. Results showed a marginally significant effect of stimulation on the Focus Index (F (1, 30) = 3.67, *p* = 0.065, η_*p*_^2^ = 0.11), with similar scores after stimulation of the DLPFC (*M* = 0.05, *SD* = 0.12) compared with after stimulation of the vertex (*M* = 0.01, *SD* = 0.13). On the other hand, there was a significant effect of stimulation on the Conflict Index (F (1, 30) = 4.19, *p* = 0.049, η_*p*_^2^ = 0.12), with higher scores after DLPFC stimulation (*M* = 0.28, *SD* = 0.12) compared with after vertex stimulation (*M* = 0.23, *SD* = 0.11). Figure 2 shows the above descriptive statistics with confidence intervals as error bars.

Results suggest that stimulation of the DLPFC impacts on the ability to deal with interference between the self and avatar perspective, and also tends to improve performance in taking the avatar perspective (relative to the self-perspective).

Further analyses were carried out on response times (accurate trials only), accuracy rates and inverse efficiency scores using 2 x 2 x 2 repeated measures ANOVAs (Stimulation: vertex x DLPFC; Consistency: consistent x inconsistent; Perspective: other x self).

### Response time (accurate trials only)

There was no main effect of stimulation (F (1, 30) = 3.47, *p* = 0.07, η_*p*_^2^ = 0.10), although there were main effects of consistency (F (1, 30) = 125.19, *p* < 0.01, η_*p*_^2^ = 0.81) and perspective (F (1, 30) = 8.82, *p* < 0.01, η_*p*_^2^ = 0.23). Specifically, longer response times were shown in the inconsistent condition (*M* = 632.81, *SD* = 113.11) compared with the consistent condition (*M* = 546.14, *SD* = 93.86) and in the self-perspective condition (*M* = 599.24, *SD* = 98.97) compared with the other perspective condition (*M* = 579.70, *SD* = 107.47).

There were interactions between stimulation and perspective (F (1, 30) = 8.13, *p* < 0.01, η_*p*_^2^ = 0.21) and between consistency and perspective (F (1, 30) = 8.64, *p* < 0.01, η_*p*_^2^ = 0.22). These were explored further using simple main effects. There were no other significant interactions.^8^

**Fig. 3:**
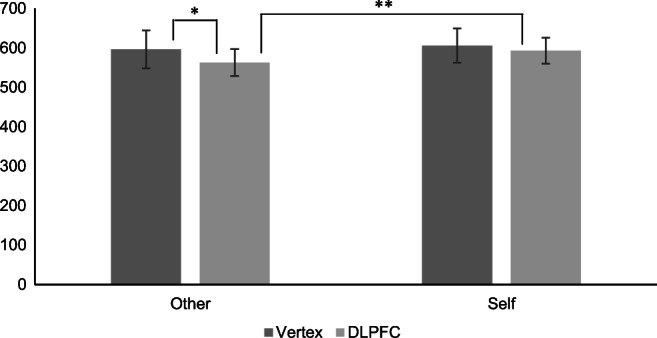
Response times to other and self-perspective trials by stimulation location (bars = confidence intervals; *p < 0.05, **p < 0.01)

For self-perspective trials, response times were not affected by stimulation of the DLPFC (*p* = 0.30). However, response times to other perspective trials were faster after stimulation of the DLPFC (compared with that of the vertex; *p* = 0.02). After stimulation of the vertex, there were no differences between self and other perspective trials (*p* = 0.25), whereas after stimulation of the DLPFC, response times to other perspective trials were significantly faster than those to self-perspective trials (*p* < 0.01; Fig. 3).

**Fig. 4:**
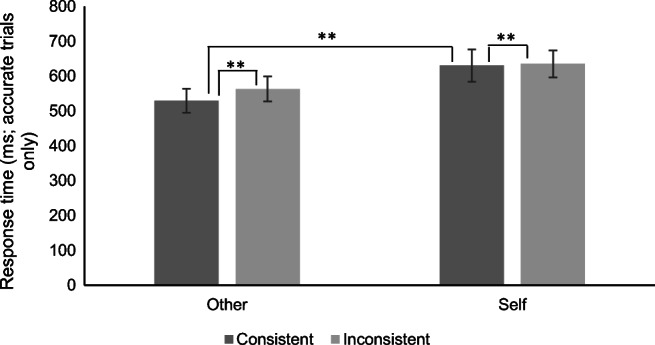
Response times by perspective and consistency (bars = confidence intervals; *p < 0.05, **p < 0*.01)*

For both self and other perspective trials, response times in the inconsistent condition were significantly longer than those in the consistent condition (both *p*s < 0.01). However, while there was no difference in response times between the perspectives in the inconsistent condition (*p* = 0.62), response times to self-perspective trials were significantly longer than those to other perspective trials in the consistent condition (*p* < 0.01; Fig. 4).

### Accuracy rates

There was a main effect of consistency (F (1, 30) = 90.11, *p* < 0.01, η_*p*_^2^ = 0.75), with higher accuracy in the consistent condition (*M* = 0.97, *SD* = 0.04) compared with the inconsistent condition (*M* = 0.89, *SD* = 0.06). There were no other main effects or interactions (*p*s > 0.11).^9^

### IES

There was a main effect of consistency (F (1, 30) = 171.69, *p* < 0.01, η_*p*_^2^ = 0.85), with worse performance in the inconsistent condition (*M* = 724.77, *SD* = 0.113.62) compared with the consistent condition (*M* = 566.56, *SD* = 151.91). There were no other main effects or interactions (*p*’s > 0.06).^10^

Analyses also were conducted on changes in egocentric and altercentric biases after DLPFC stimulation (compared to vertex stimulation) for response times, accuracy rates, and IES. These showed no significant differences from zero (see Appendix A).

## Discussion

The purpose of this paper was to provide a first step towards exploring the role of the DLPFC in VPT. While the rTPJ (Schurz et al., 2015) and the right prefrontal cortex (Beck, Rossion & Samson, 2018), have hitherto been the foci of much perspective-taking research, the current findings extend the brain area network that appears to be involved (see also Bukowski, 2018). Indeed, in line with predictions, findings suggest that self-other interference, as measured by the conflict index, may be causally related to the rDLPFC. Specifically, inhibition of the rDLPFC resulted in an overall increase in the magnitude of the conflict index compared with inhibition of the vertex (control condition), with the focus index relatively unaffected. In other words, stimulation of the DLPFC appeared to create greater difficulties when dealing with competing perspectives.

Analyses of response times showed that stimulation of the DLPFC resulted in faster processing of “other” perspective trials in a pattern that is typical for L1 VPT research. Research has suggested that calculation of the other perspective in the L1 VPT is at least relatively automatic (Qureshi, Apperly & Samson, 2010). It is therefore possible to speculate that stimulation of the DLPFC increases the processing speed of the (automatic) calculation of the other perspective. Accuracy and IES on the other hand, do not appear to be affected (with only a main effects of consistency shown).

The DLPFC has been associated with cognitive control, particularly with task switching (Badre & Wagner, 2006), memory updating (Edin et al., 2009), response sequencing, monitoring, and manipulation (Kim et al., 2013; Owen et al., 1996). As such, the DLPFC may be suggested to have a domain-general role in selection. With respect to the response time analyses, impairing the ability to monitor and manipulate responses may result in the automatically calculated other perspective becoming more salient. The IFG also has been cited as key to self-other interference (Hartwright, Hansen & Apperly, 2016a) and has been associated with inhibitory control (Aron et al., 2003, 2004, 2014; Chambers et al., 2006). Amalgamating existing research with the current results, we therefore tentatively suggest that these neural areas may be causally related with both self-other interference, as well as nonsocial inhibitory control, perspective-taking may be (at least in part) domain-general, whereas the automatic calculation of the other perspective may be more domain-specific (although see Samson, Houthuys & Humphreys (2015) regarding self-perspective inhibition and executive control).

Self-other interference may then rely on both response monitoring and manipulation (DLPFC), as well as inhibitory control (IFG). Specifically, it has been suggested that participants initially and automatically calculate the “other” perspective (McCleery et al., 2011; Qureshi, Apperly & Samson, 2010), whereas perspective selection occurs much later, after responses from both the self and (automatically calculated) other perspectives have been initiated (Qureshi et al., 2019). The processes involved in arriving at perspective selection may thus be posited to rely on the DLPFC (response monitoring and perhaps manipulation, as well as working memory; Qureshi & Monk, 2018), while perspective selection itself is reliant on inhibitory control (Hartwright, Hansen & Apperly, 2016a; Qureshi, Apperly & Samson, 2010). Indeed, the fact that the DLPFC and IFG are linked to the Fronto-Parietal Control Network (related to volitional attention) and Default Mode Network (also including bilateral TPJ and mPFC, associated with reflexive attention), respectively, gives further evidence that they may have different roles within VPT (Hartwright et al., 2016a, b; Samson et al., 2015; Yeo et al., 2011).

### Limitations and future directions

The order of sessions was randomised between participants to minimise practice effects and the nature of the task (which presented a large number of trials and required relatively fast response times) would suggest that it would be difficult to recall specific trials and responses. Indeed, the error rates between first and second sessions did not significantly differ, where a practice effect would be seen if the error rate was significantly lower in the second sessions. Nevertheless, the current results should be viewed in light of the potential limitations of within-subject testing, caused by possible practice or familiarity effects caused by repeat testing. Post-hoc power analyses suggest that the power to detect the observed effect sizes was 0.97 (Faul et al., 2009), although a more well powered and pre-registered study (while the study was not preregistered (Munafò et al., 2017), the data are available on request) should be conducted in order to enable stronger inferences and conclusions (Zwaan et al., 2017).

Potential interindividual variability in the effects of cTBS (Huang & Mouraux, 2015; Paracampo, Pirruccio, Costa, Borgomaneri, & Avenanti, 2018) should be borne in mind when considering the current research. For example, it has been suggested that self-reported dispositional cognitive empathy predicted whether cTBS of rTPJ enhanced rather than impaired performance in the self-other distinction of empathy (Bukowski, Tik, Silani, Ruff, Windischberger, & Lamm, 2019). Our data indicated that the majority of participants showed increases in focus (18/31) and conflict (19/31) indices following cTBS, suggesting that CTBS effects were fairly consistent and thus there is not substantial cause to suspect underlying variability in participants’ baseline capacity. Nevertheless, self-reported differences in skills, such as perspective taking and/or empathy, cannot be excluded. Future research would therefore benefit from the inclusion of baseline assessments of such individual differences (e.g., by gauging Interpersonal Reactivity Indices before testing) to provide greater assurances.

Measures of executive function such as working memory (e.g., simple or complex span tasks) and inhibitory control (Stroop, anti-saccade or stop-signal tasks) could be used alongside the L1 VPT task. This would allow us to ascertain precisely which aspects of executive function also are impacted by disruption of the rDLPFC, and hence may be directly linked to L1 perspective-taking. To add further weight to the domain-general role of the rDLPFC in L1 VPT, future research also may consider the addition of a further control condition. Specifically, this condition should not require perspective-taking but still necessitate conflict or interference resolution, in conjunction with stimulation of the rDLPFC. This would allow researchers to ascertain whether interference in that control condition also was impaired by rDLPFC stimulation, as would be expected.

Research suggests that egocentric interference is shown for both L1 and L2 judgments (with this interference larger for L2 judgments), while unintentional perspective taking was only present in L1 judgments (Surtees, Samson & Apperly, 2016). Future research may therefore benefit from the additional inclusion of level-2 VPT tasks. From the current findings, it may be speculated that the DLPFC would increase egocentric interference for both L1 and L2 judgments, although the magnitude of this increase would be expected to be greater for L2 judgments. On the other hand, it would be anticipated that the automatic perspective-taking shown in L1 judgments would be relatively unimpaired. Whilst the current study stimulated the rDLPFC, neuroimaging studies on VPT have found activation of the left middle prefrontal gyrus (Ramsey et al., 2013; Schurz et al., 2013). Further evidence has shown that while the rTPJ may be related to inhibition and also perspective selection, the lTPJ may be involved in belief-attribution (see Mahy, Moses & Pfeiffer, 2014). It therefore also is suggested that the lateralisation of areas associated with self/other interference and perspective calculation be investigated in the future.

## Conclusions

Results suggest that the DLPFC, responsible for response monitoring and manipulation, is involved in self-other interference in a L1 VPT task. This provides potential evidence that VPT is (at least in part) domain-general, although perspective calculation may be domain-specific.

### Open Practices Statement

The data and materials for all experiments are available from the lead author. The study was not preregistered.

## Appendix A

Egocentric and altercentric biases

Changes in egocentric bias (inconsistent – consistent other perspective) and altercentric bias (inconsistent – consistent self perspective) after stimulation of the DLPFC relative to after vertex stimulation were calculated for response times, accuracy rates and IES (see Table 1).

**Table 1:** One-sample t-tests (theoretical value = 0) for egocentric and altercentric bias changes after DLPFC stimulation (compared to vertex stimulation)

Measure	Bias	t-test	*p*	Mean difference	(95% confidence interval of the difference)
RT	Egocentric	t (30) = .00	1.00	-.01	-27.45 – 27.44
	Altercentric	t (30) = .94	.36	12.09	-14.22 – 38.41
Accuracy Rate	Egocentric	t (30) = -1.50	.15	-.03	-.07 - .01
	Altercentric	t (30) = -.50	.62	-.01	-.05 - .03
IES	Egocentric	t (30) = 1.01	.32	23.02	-23.74 – 69.78
	Altercentric	t (30) = 1.09	.27	25.01	-21.83 – 71.86

## Footnotes

1 Patients had lesions in frontal and nonfrontal regions and were further classified to assess the general frontal effect of frontal damage into four groups: frontal, nonfrontal (and control). These were further divided by site of lesion into right frontal, left frontal, bifrontal, right nonfrontal, and left nonfrontal groups. Each discrete frontal legion (Stuss et al., 1995, 2000) also was scored for presence or absence of any lesion, and correlations of performance with these more specific areas were completed. For visual-perspective taking, a general frontal effect was shown, with a more important role for the right frontal region. However, no correlations were shown with more localised regions.

2 Defined as follows: cognitive theory of mind requires cognitive understanding of the difference between the self and others knowledge, measured by (for example) the false belief task (so knowledge about beliefs); affective theory of mind also requires an understanding of the others emotional state (knowledge about emotions; Kalbe et al., 2010)

3 Bukowski and Samson (2017) have derived separate measures from this task: “conflict,” relating to the interference between the self and other perspectives, and “focus,” the relative ease of judgments relating to self versus other perspectives (see Fig. 1; Focus is calculated by self-perspective – other perspective trial performance. Conflict is derived from inconsistent perspective – consistent perspective trial performance).

4 The other combination possible of an effect of stimulation on the focus but not the conflict index is possible, but unlikely considering available evidence.

5 This is similar to other TMS studies, e.g., Sagliano et al., 2016; Ferrari et al., 2017.

6 Research has suggested that using the active motor threshold (AMT) may increase the excitability of the spinal cord rather than the motor cortex (Fried et al., 2019; Rossini et al., 2015), meaning that using the resting motor threshold (RMT) may be better for cortical brain stimulation. This approach is akin to that taken by similar research in the area of higher cognitive functions (Jung & Ralph, 2016).

7 Use of IES is not recommended if speed-accuracy trade-offs are shown (indicated by no positive correlation between response times and error rates) and if the average error rate is above 0.10 (see Bukowski & Samson, 2017). The correlation between response time and error rate was positive in both stimulation conditions (control: *r* (30) = 0.42, *p* < 0.01; active: r (30) = .32, *p* < 0.01), and the mean error rate across stimulation conditions was less than 0.10 (control: 0.08 (SD = 0.11). active: 0.07 (SD = 0.09), meaning both recommendations for using IES were met.

8 Stimulation x consistency: F (1, 30) = 0.31, *p* = 0.58, η_*p*_^2^ = 0.01); Stimulation x consistency x perspective: F (1, 30) = 0.66, *p* = 0.42, η_*p*_^2^ = 0.02).

9 Stimulation: F (1, 30) = 0.16, *p* = 0.69, η_*p*_^2^ = 0.01; Perspective: F (1, 30) = 0.00, *p* = 0.99, η_*p*_^2^ = 0.00; Stimulation x Consistency: F (1, 30) = 2.76, *p* = 0.11, η_*p*_^2^ = 0.08; Stimulation x Perspective: F (1, 30) = 0.02, *p* = 0.90, η_*p*_^2^ = 0.00; Consistency x Perspective: F (1, 30) = 0.04, *p* = 0.84, η_*p*_^2^ = 0.00; Stimulation x Consistency x Perspective: F (1, 30) = 0.38, *p* = 0.54, η_*p*_^2^ = 0.01.

10 Stimulation: F (1, 30) = 1.72, *p* = 0.20, η_*p*_^2^ = 0.05; Perspective: F (1, 30) = 1.79, *p* = 0.19, η_*p*_^2^ = 0.06; Stimulation x Consistency: F (1, 30) = 2.65, *p* = 0.11, η_*p*_^2^ = 0.08; Stimulation x Perspective: F (1, 30) = 3.83, *p* = 0.06, η_*p*_^2^ = 0.11; Consistency x Perspective: F (1, 30) = 1.95, *p* = 0.17, η_*p*_^2^ = 0.06; Stimulation x Consistency x Perspective: F (1, 30) = 0.00, *p* = 0.96, η_*p*_^2^ = 0.00.
